# Trends of fear and anger on YouTube during the initial stage of the COVID-19 outbreak in South Korea

**DOI:** 10.1186/s12889-024-19023-6

**Published:** 2024-06-04

**Authors:** Jae-Joon Lee, Jongwoo Kim, Soo-Kyoung Lee

**Affiliations:** 1 Sookmyung Research Institute of Humanities, Sookmyung Women’s University, 100 Cheongparo 47 gel, Yongsan-gu, Seoul, 04310 South Korea; 2 BK21Four Program, Department of Sociology, Yonsei University, 3-101, 84 Mapo-daero 11 gil, Mapo-gu, Seoul, 04133 South Korea; 3 Seoul National University, Bigdata Convergence and Open Sharing System 1, Gwanak-ro, Gwanak-gu, Seoul, 08826 South Korea

**Keywords:** Anger, COVID-19, Fear, Pandemic, YouTube, Trends

## Abstract

**Background:**

The COVID-19 pandemic has been the most widespread and threatening health crisis experienced by the Korean society. Faced with an unprecedented threat to survival, society has been gripped by social fear and anger, questioning the culpability of this pandemic. This study explored the correlation between social cognitions and negative emotions and their changes in response to the severe events stemming from the COVID-19 pandemic in South Korea.

**Methods:**

The analysis was based on a cognitive-emotional model that links fear and anger to the social causes that trigger them and used discursive content from comments posted on YouTube’s COVID-19-related videos. A total of 182,915 comments from 1,200 videos were collected between January and December 2020. We performed data analyses and visualizations using R, Netminer 4.0, and Gephi software and calculated Pearson’s correlation coefficients between emotions.

**Results:**

YouTube videos were analyzed for keywords indicating cognitive assessments of major events related to COVID-19 and keywords indicating negative emotions. Eight topics were identified through topic modeling: causes and risks, perceptions of China, media and information, infection prevention rules, economic activity, school and infection, political leaders, and religion, politics, and infection. The correlation coefficient between fear and anger was 0.462 (*p* < .001), indicating a moderate linear relationship between the two emotions. Fear was the highest from January to March in the first year of the COVID-19 outbreak, while anger occurred before and after the outbreak, with fluctuations in both emotions during this period.

**Conclusions:**

This study confirmed that social cognitions and negative emotions are intertwined in response to major events related to the COVID-19 pandemic, with each emotion varying individually rather than being ambiguously mixed. These findings could aid in developing social cognition-emotion-based public health strategies through education and communication during future pandemic outbreaks.

## Background

COVID-19 is a large-scale infectious health crisis that occurred accidentally, spread rapidly, and lasted for a long time. To respond effectively to such events, public healthcare workers and leadership need to consider social emotions and cognitive attitudes that have not been paid attention to.

Vaccination and social isolation are essential components of quarantine policy. Early diagnosis, vaccination, and treatment are important aspects of infectious disease policy [1], but they were not effective in emergencies where COVID-19 suddenly occurred on a large scale. For this reason, healthcare measures centered on different degrees of social isolation were prioritized, with varying degrees of effectiveness in different countries.

In South Korea, social isolation was relatively strong in the early stages of the outbreak due to public fear, and healthcare policies such as social distancing, infection testing, and voluntary quarantine were successfully implemented as social isolation was gradually relaxed [2, 3]. In countries that implemented stronger quarantine policies, there was significant citizen resistance [4, 5]. Meanwhile, public awareness of the COVID-19-related events in Daegu, South Korea, generated social outrage, leading to support for stronger quarantine policies and increased criticism of coercive quarantine policies [6].

To effectively implement COVID-19 quarantine policies, the active participation of citizens was necessary [7, 8], and healthcare professionals needed to continuously monitor and consider negative emotions and cognitive attitudes toward COVID-19-related events. Health campaigns encouraging citizen participation were introduced to mitigate psychological stress and resistance [9, 10]. The change in citizens’ social cognition and emotion during COVID-19 was largely driven by the surge in social network service use due to prolonged social isolation [11].

Our study analyzed changes in citizens’ social emotions and cognitive attitudes toward events that occurred during the first year after the COVID-19 outbreak in South Korea, focusing on social networks, especially YouTube comments. While many studies focused on individuals’ negative emotional states during COVID-19 [12, 13], we emphasize that healthcare actions are not just determined by personal emotional and cognitive experiences but are more driven by social judgments formed by cognitive evaluations of information and emotions about relevant events [14–16]. We specifically looked at the correlation between socio-cognitive appraisals and affective changes in response to COVID-19-related events in South Korea.

Cognitive appraisals in the early stages of the COVID-19 outbreak in South Korea were influenced by the lack of clear information, ethical blame on groups violating hygiene rules, and accusations of religious or political bias, which affected emotions of fear and anger.

Our study explains how social cognitive judgments and emotional changes are influenced by social network-based communication during a large-scale infectious disease outbreak, and suggests that understanding citizens’ social cognition and emotions in healthcare can help drive effective problem-solving.

### Literature review

The cognitive-emotional theory suggests that the reciprocal impact between cognitive judgments and emotions plays a significant role in social decision-making [17]. In social crises like COVID-19, negative emotions such as anxiety and anger are prevalent. However, many healthcare policies focus solely on the rational cognitive judgment of information to address issues. Social threats can lead to widespread negative emotions in communities, making rational decision-making challenging. Studies have highlighted how fear and anger affect social decision-making during pandemics [18, 19] or how they are influenced by cognitive assessments of negative social situations [20]. Social emotions are psychological reactions that stem from the cognitive evaluation of situations that individuals and groups encounter [21–23]. Furthermore, changes in the negative emotions experienced by citizens, such as fear and anger, can be identified by their cognitive evaluation of the events they encounter [21, 24–27]. Cognitive appraisals, particularly, depend on the gain/loss dynamics of the parties in the event and their ethical evaluations. Fear arises from a specific or ambiguous threat that induces distress. Anger is triggered when the consequences of an event are viewed as ethically troubling by those involved. Anger may be expressed as a substitute for social response, even if it does not directly affect oneself [26].

Amid the COVID-19 pandemic, communication via social networks indirectly influenced how people perceived events, shaping their emotions [11, 28–30]. Healthcare professionals have emphasized the importance of cognitions and emotions. However, during the social panic caused by COVID-19-related events, negative emotions were grouped as vague mixed emotions [31, 32], or only the effects of specific negative emotions were described [33]. This limits the detailed understanding of the impact of social cognitions and emotions. Meanwhile, it is important to consider social cognitive evaluations of events related to COVID-19 and the development of different emotions in response to these events. By doing so, we can assess how negative emotions affect healthcare decision-making.

Based on this theoretical background, our study analyzed the correlation between social cognitions and emotions in response to major events that occurred during the first year of COVID-19 in South Korea. Our study is distinct from previous research focusing solely on citizens’ cognitive factors or emotions during a pandemic quarantine. Unlike studies that have vaguely explained negative emotions, we explain the attributional factors based on citizens’ social cognitive evaluation of events related to COVID-19. Additionally, we clarify the changes in trends regarding distinguishable anxiety and anger.

## Materials and methods

### Data collection

YouTube comments document user reactions to videos on the platform. For our study, we focused on videos related to COVID-19 and gathered comments in Korean. We used search terms like ‘COVID-19’, ‘COVID’, and ‘Corona’. The videos were accessed through the YouTube API, with researchers pulling data by entering specific keywords. Although the API can provide up to 500 videos, we chose to focus on the top search results. YouTube’s search ranking algorithm prioritizes relevance. While specifics of the algorithm are not public, previous research suggests that factors such as titles, tags, descriptions, and video content influence the ranking [34]. The search results were sorted based on relevance using the YouTube search algorithm. Between January and December 2020, 182,915 comments were gathered from 1,200 videos by selecting the top 100 most relevant videos each month. Our corpus consisted of 14,506 terms, with a total term frequency of 583,947.

### Data processing

The data collected consisted of Korean texts, which were translated into English for analysis. The translation was done using the Google Translate API. Following translation, text pre-processing was carried out. The first step involved text tokenization, which included part-of-speech (POS) tagging, normalization, and stemming. Next, a term extraction process was conducted to create a corpus by extracting relevant vocabulary for analysis. The corpus was created by extracting nouns using a morpheme analyzer. The decision to focus on nouns in this research was based on existing studies that showed nouns alone can reflect emotions and attitudes in large datasets [35, 36]. Additionally, stop words were eliminated by removing unneeded terms and by processing synonyms with a dictionary.

### Data analysis

A term-document matrix (TDM) was created to calculate the co-occurrence matrix between the documents and terms. Each comment was treated as a document, with the terms being nouns within the comments that were assessed during the initial pre-processing. Following this, a topic model analysis employing the Latent Dirichlet allocation (LDA) algorithm was used to uncover potential topics present within the documents. The optimal number of topics (k = 8) for the analysis was determined by assessing coherence. This determination was made by comparing models with a minimum of five topics and a maximum of 20 topics. The criteria for evaluating the model included an alpha value of 0.02 and a beta value of 0.01. The terms within each topic were arranged in descending order of probability. The qualitative coding methodology used in this study was Grounded Theory, as specified [37]. The process involved three researchers conducting cross-coding in the following order: (1) Initial coding: Identifying core concepts or characteristics of each topic derived from the topic model, based on representative words of each topic, without preconceived units of meaning. (2) Focused coding: Analyzing relationships between the identified concepts or characteristics to organize them into broader categories and establishing abstract concepts. (3) Sorting and integrating: Synthesizing thematic categories to refine abstract concepts into specific terms used as labels for the topics. Researchers were allowed to repeat coding steps to verify and adjust labels as needed during the analysis process.

Network visualization was conducted using a co-occurrence matrix. To analyze the semantic network, a 1-mode network was established from the co-occurrence matrix, with a connection range of 1 (network window = 1). The OpenOrd force-directed graph drawing algorithm was employed for network visualization, utilizing Netminer 4.0 and Gephi for analysis and visualization.

For emotion analysis, the emotion score of each term, determined during pre-processing, was examined using the National Research Council (NRC) Word-Emotion Association Lexicon emotion dictionary. This lexicon includes English terms and their connections to eight core emotions (anger, fear, anticipation, trust, surprise, sadness, joy, and disgust), as well as two emotional valences (negative and positive).

**Fig. 1:**
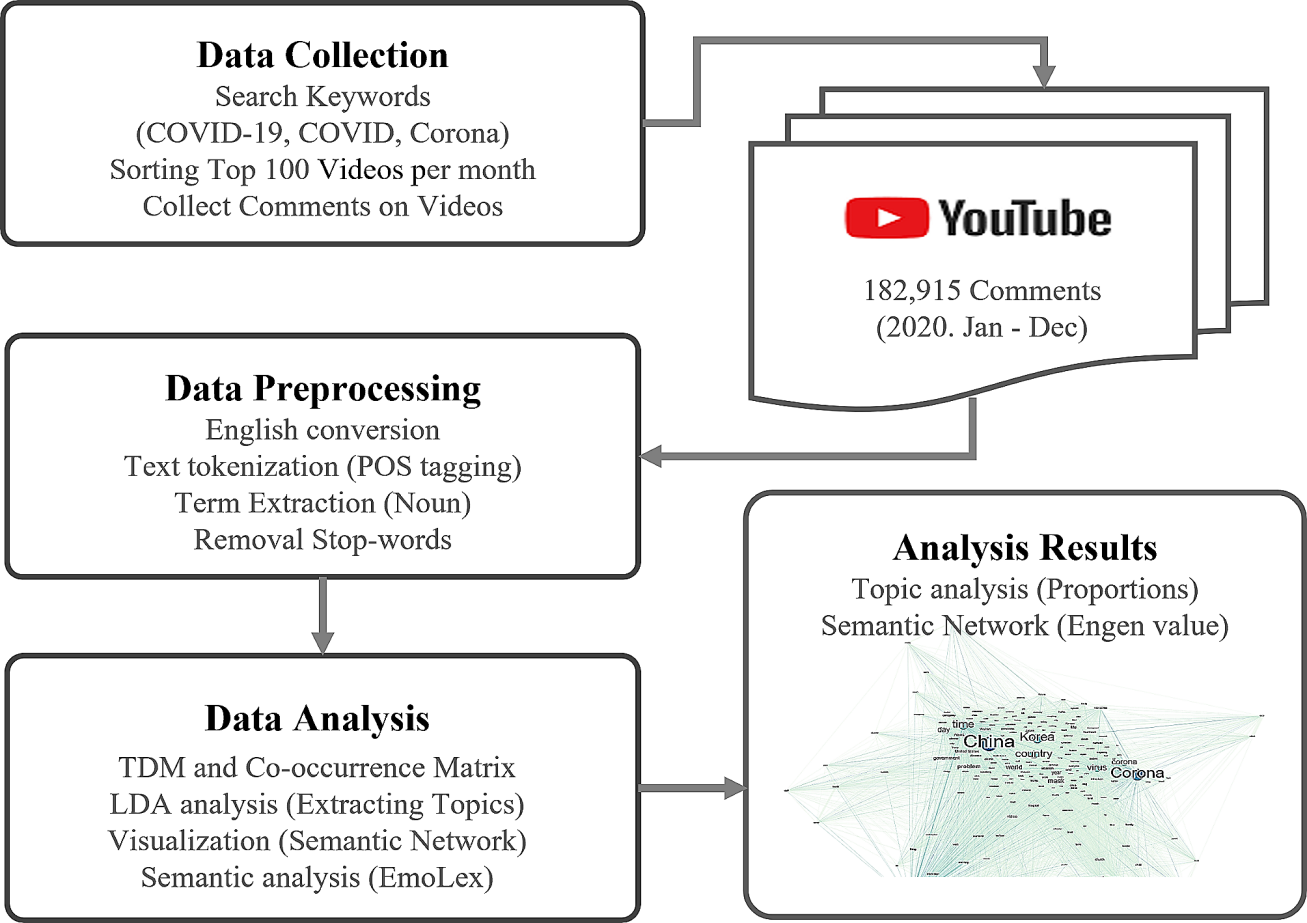
Process of data collection and analysis

The NRC Word-Emotion Association Lexicon [38] has been translated into 108 foreign languages as of August 2022. Though there may be errors and cultural diversity in the machine-translated results, the lexicon notes that emotional vocabulary can be consistently applied in various foreign languages. Additionally, it has been used in Korean emotion analysis [39, 40]. The research team considered these factors and utilized the NRC Emotion Lexicon results translated from English to Korean. Emotion scores were assigned to each comment term. The daily average value of the assigned scores was calculated. Pearson’s correlation coefficient was used to confirm the correlation between anger and fear trends over time based on the average emotion score. Figure 1 depicts the data collection and analysis process.

### IRB approval

The study was approved by the Institutional Review Board of the Sookmyung Women’s University (IRB No: SMWU-2304-HR-015).

## Results

We extracted 182,915 comments from 1,200 videos collected between January and December 2020. After tokenizing the comments and converting them to English, we identified the top 20 keywords for COVID-19-related video comments in this study. Table 1 demonstrates the frequency of each keyword and degree centrality, which measures the degree to which one keyword is connected to other keywords. The most frequently searched keywords were ‘person’, ‘country’, ‘virus’, ‘time’, and ‘mask’. Geographically, the keywords were associated with ‘China’, ‘Korea’, ‘United States’, ‘Wuhan’, and ‘Japan’.

**Table 1:** Frequency and degree centrality of the top 20 keywords in the general and geographic division

No.	General keywords	Frequency	Degree Centrality	No.	Geographic keywords	Frequency	Degree Centrality
1	person	22,109	1,874	1	China	10,666	1,488
2	country	8,321	1,067	2	Korea	7,073	1,240
3	virus	7,482	1,101	3	United States	2,377	530
4	time	7,095	1,107	4	Wuhan	2,243	509
5	mask	6,361	825	5	Japan	1,703	437
6	a lot of	5,632	1,166	6	Earth	1,199	205
7	corona	5,081	896	7	Chinese	1,007	337
8	day	4,894	889	8	North Korea	886	258
9	world	4,075	825	9	Daegu	852	245
10	year	3,659	668	10	Moon	722	178
11	government	3,506	664	11	Seoul	673	290
12	video	3,163	627	12	Europe	387	131
13	vaccine	2,947	490	13	World	343	148
14	problem	2,618	745	14	Australia	296	120
15	life	2,592	621	15	Germany	293	116
16	news	2,588	553	16	Hong Kong	261	124
17	money	2,478	449	17	Busan	207	101
18	staff	2,207	225	18	Taiwan	203	84
19	school	2,193	421	19	Shincheonji	192	80
20	situation	2,043	384	20	Vietnam	182	84

In the context of emotional values in YouTube comments, anger scored an average of 0.355 points (range 0.308–0.392), while anxiety scored higher with an average of 0.592 points (range 0.509–0.708). A correlation coefficient of 0.462 (*p* < .001) was found between fear and anger, indicating a strong relationship between these emotions. This strong correlation between fear and anger further supported the connection between the two emotions.

**Fig. 2:**
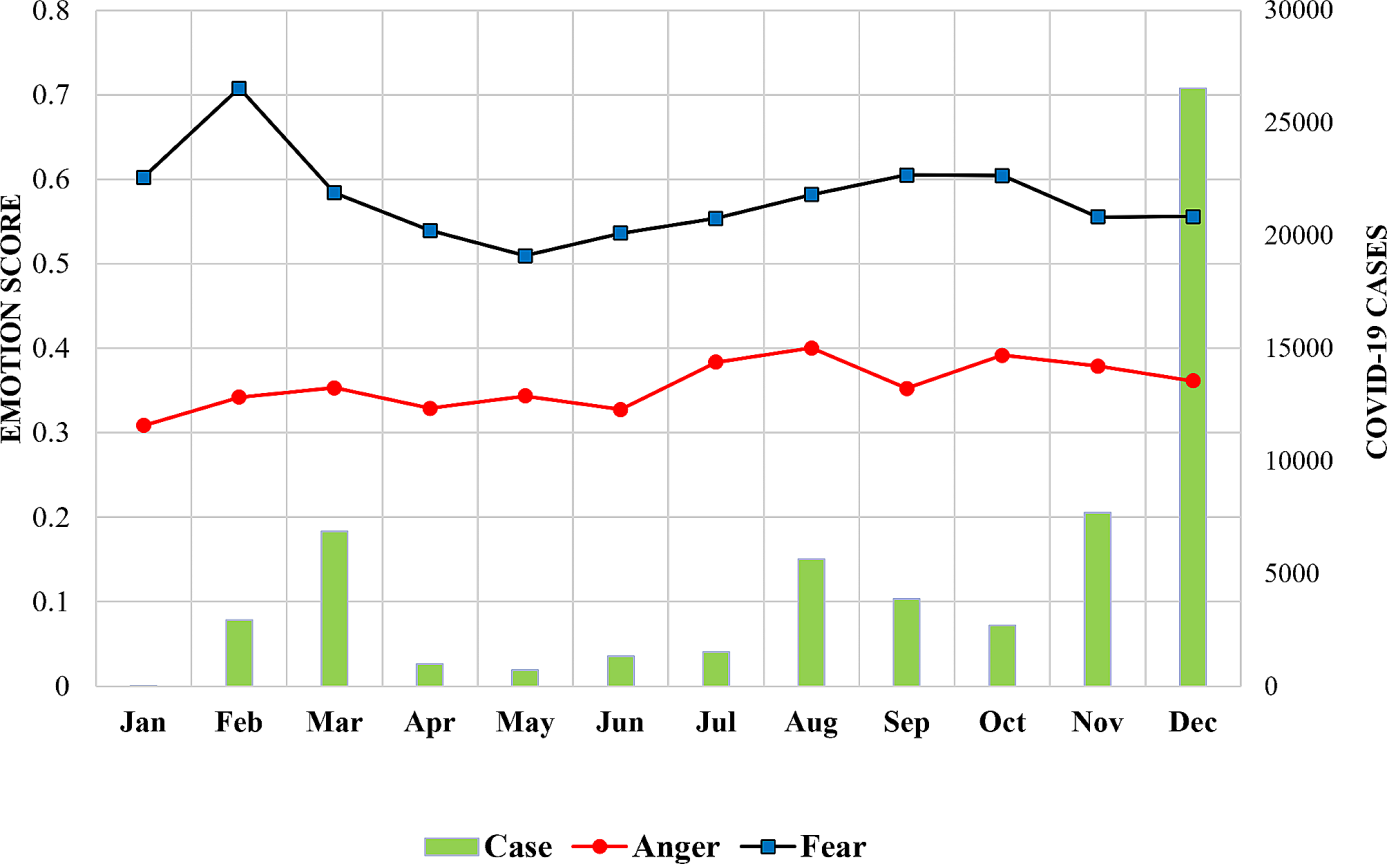
Emotional trends and COVID-19 cases (January 2020–December 2020)

Changes in fear and anger over time indicated a connection between the two emotions. Three patterns of monthly emotional trends were identified. Fear consistently appeared stronger than anger throughout all periods. In January, the early days of the pandemic, fear and anger were expressed at the highest levels, with a pattern of increase starting in August. Fear experienced a sharp increase during the initial stages of the pandemic, while anger saw a slight increase post-February. Furthermore, fear decreased and reached its peak in August. The most significant increase in fear was observed during the early pandemic stages, followed by a decrease in November and December as confirmed cases rose rapidly (see Fig. 2).

**Fig. 3:**
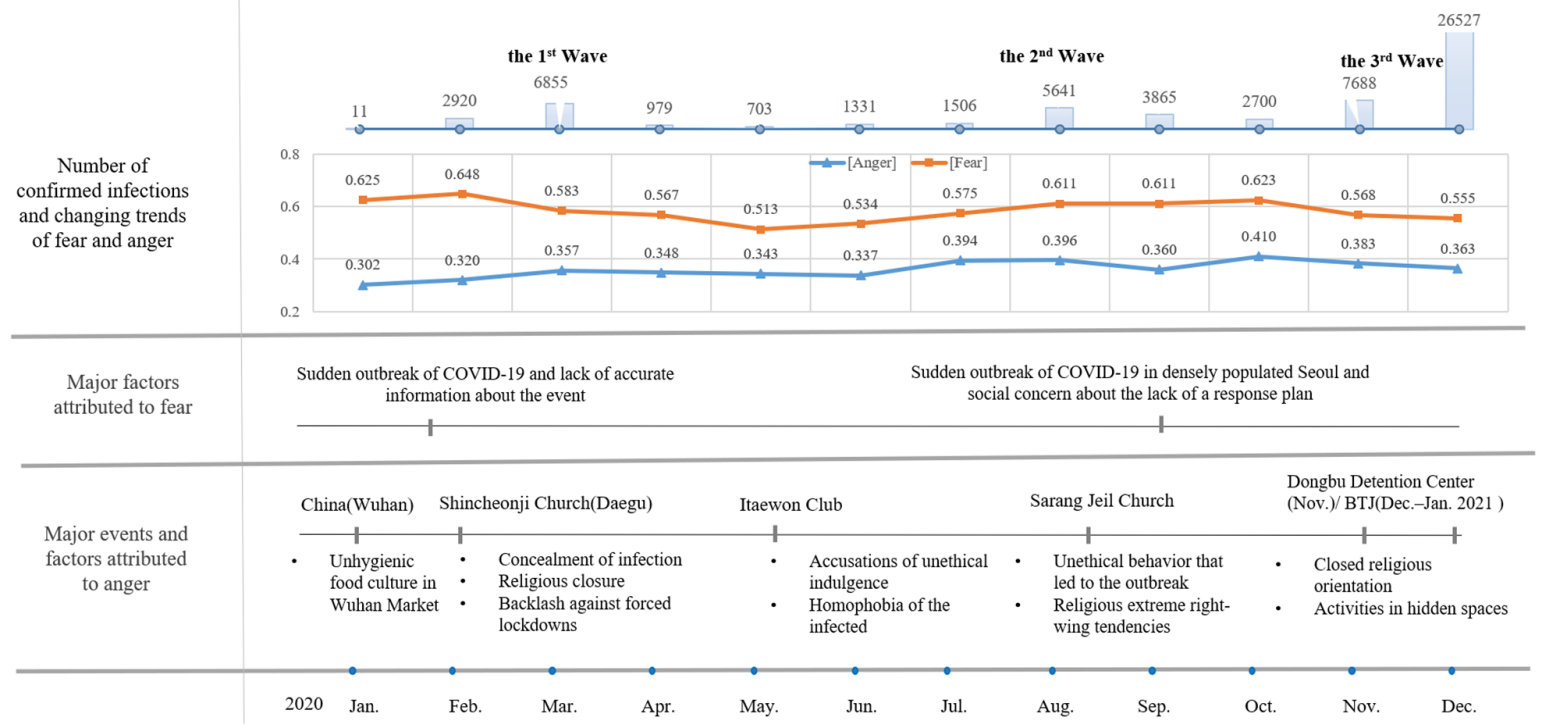
Changes in fear and anger during the first year of the COVID-19 pandemic (2020). The figure describes three things: The number of confirmed infections and changing trends of fear and anger, major factors attributed to fear, and major events and factors attributed to anger. [Top of figure] In the first year of the COVID-19 outbreak in South Korea, there were three waves of spikes in infections. In the graph, the number of cases and the intensity of emotions are changing. The emotion value is the intensity of the emotion averaged over each month, from a minimum of 0 to a maximum of 1. The peaks of the waves do not show the peak increases in social negative emotions, namely anxiety and anger. It also shows that the increase in anxiety is not proportional to the increase in anger. The graph illustrates that anxiety and anger are driven by different attributional factors. [Middle of figure] The cognitive attribution of social anxiety is the lack or ambiguity of information regarding COVID-19, and social anxiety shows the highest increase in the early stages of the outbreak. There is also a modest increase in social fear when the political protests at Sarang Jeil Church are resolved (September) and just before the third wave begins to rise (October). [Bottom of figure] Anger arises from cognitive appraisals of major events. Social anger peaks with negative appraisals of all aspects of ethics, religion, and politics, especially during the protests of Sarang Jeil Church’s leader and his followers showing their religious and political bias (August)

Fear and anger fluctuated based on the associated topic, influenced by the triggering events and contextual factors. The topic analysis revealed that citizens reacted most strongly to social events related to COVID-19, such as the large-scale outbreak in Wuhan, China (January), the Daegu Shincheonji religious outbreak (February and March), the Itaewon club outbreak (May), the Sarang Jeil Church and Gwanghwamun political rally outbreaks (August), the Dongbu Detention Center Mass Outbreak (November), and subsequent religious outbreaks like the Inter-Coop Mission at the Back to Jerusalem (BTJ) Center (December 2020–January 2021) and the International Mission (January 2021). These events contributed to distinguishing between the first wave (February to early May), second wave (mid-August to mid-October), and the third wave (mid-November to end-January 2021) of COVID-19 (Fig. 3).

**Fig. 4:**
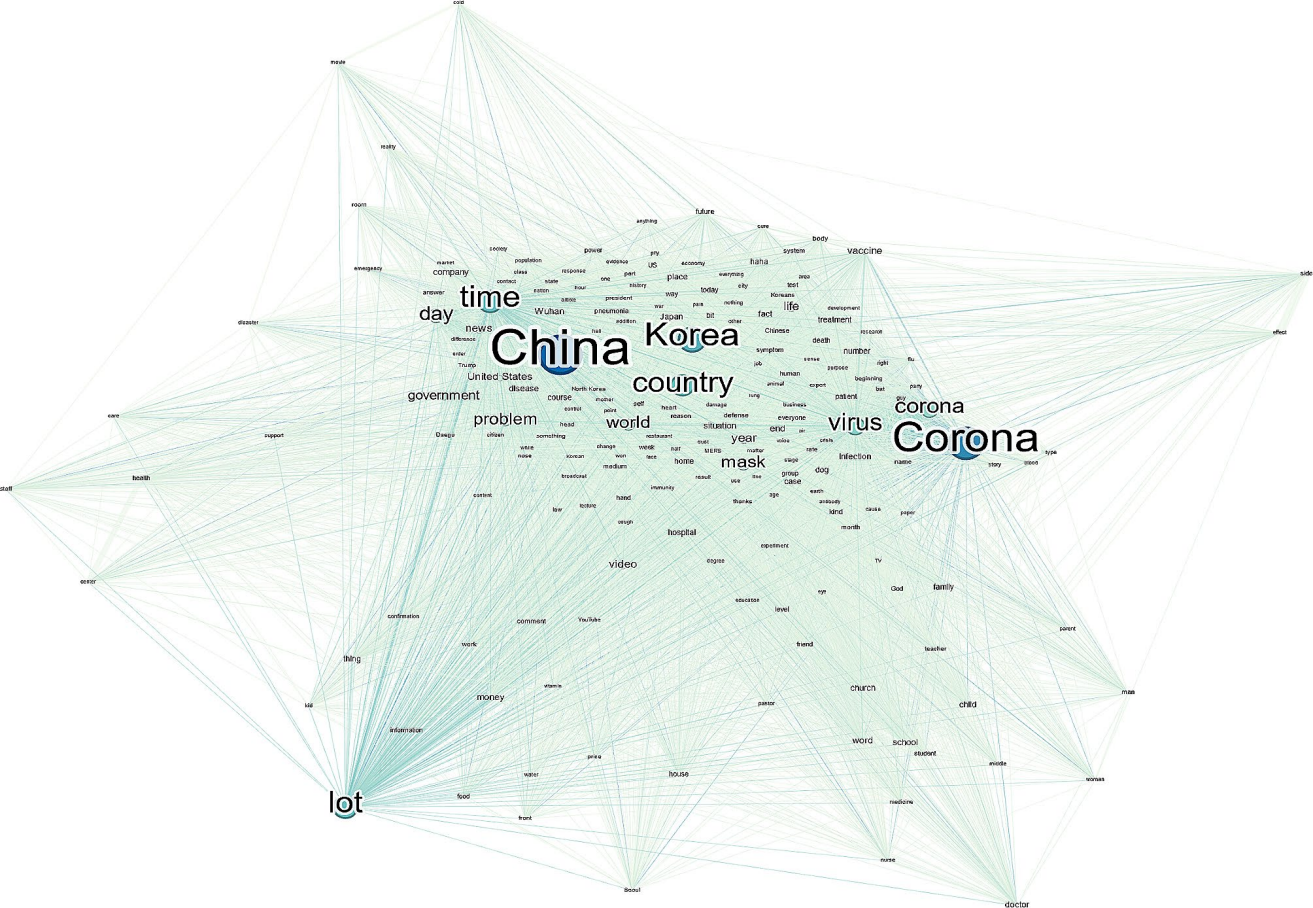
Keyword network of comments related to COVID-19 in YouTube videos

We visualized the keyword network using a co-occurrence matrix of comments on COVID-19-related YouTube content (Fig. 4). Through topic modeling, we identified eight topics: causes and risks (21%), perceptions of China (14%), media and information (13%), infection prevention rules (12%), economic activity (12%), school and infection (10%), political leaders (10%), and religion, politics, and infection (9%).

The top 30 keywords for each topic are listed in Table 2. We acknowledge that assigning topic labels involves a qualitative interpretation process and may be subject to the researcher’s subjectivity. To enhance the validity of the labels, multiple researchers independently assigned labels and then compared and adjusted them through discussion. However, we recognize that seeking input from external experts could have further strengthened the label selection process, which is an area for improvement in our study. To address this limitation and provide more transparency, we have included the probability of each constituent word within the topics.

**Table 2:** Top 30 keywords in YouTube comments about COVID-19

Topic	Labeling	Proportion	Terms (Probability)
1	Causes and risks of infectious disease	0.21	virus (0.057) Corona (0.026) vaccine (0.025) China (0.015) time (0.013) flu (0.012) year (0.011) treatment (0.01) bat (0.01) rate (0.01) disease (0.009) Wuhan (0.009) infection (0.008) effect (0.008) human (0.007) animal (0.007) death (0.007) world (0.007) earth (0.007) mortality (0.006) side (0.006) body (0.006) Korea (0.006) problem (0.006) immunity (0.006) pneumonia (0.006) lot (0.005) antibody (0.005) country (0.005)
2	Perception of China	0.14	China (0.083) country (0.06) Korea (0.04) world (0.024) United States (0.017) Corona (0.014) Japan (0.013) virus (0.013) government (0.011) Wuhan (0.01) time (0.01) entry (0.009) North Korea (0.008) lot (0.007) problem (0.007) situation (0.006) corona (0.006) day (0.006) Koreans (0.006) Chinese (0.005) dust (0.005) place (0.005) vaccine (0.005) population (0.005) thing (0.005) war (0.005) money (0.004) death (0.004) US (0.004) year (0.004)
3	Media and information on COVID-19	0.13	video (0.024) staff (0.023) Corona (0.022) lot (0.022) time (0.02) doctor (0.012) information (0.011) health (0.01) life (0.01) day (0.01) corona (0.009) everyone (0.009) mask (0.008) news (0.007) Korea (0.007) teacher (0.007) nurse (0.007) comment (0.007) country (0.007) virus (0.007) word (0.006) work (0.006) situation (0.006) Dr (0.006) patient (0.006) lecture (0.005) care (0.005) song (0.005) thanks (0.005) world (0.005)
4	Infection prevention rules	0.12	mask (0.046) day (0.025) time (0.015) symptom (0.014) lot (0.014) hand (0.014) virus (0.014) corona (0.014) Corona (0.011) home (0.011) hospital (0.01) nose (0.01) body (0.008) water (0.007) house (0.007) cough (0.007) week (0.006) fever (0.006) air (0.006) pain (0.006) infection (0.006) patient (0.006) room (0.005) heat (0.005) diarrhea (0.005) degree (0.004) vitamin (0.004) school (0.004) blood (0.004) today (0.004)
5	Economic activity and COVID-19	0.12	country (0.016) money (0.015) year (0.013) government (0.013) time (0.012) Korea (0.012) lot (0.012) Corona (0.011) company (0.009) problem (0.008) life (0.008) end (0.007) world (0.007) month (0.007) China (0.007) self (0.006) day (0.006) mask (0.006) church (0.005) number (0.005) corona (0.005) price (0.005) business (0.005) place (0.005) rate (0.005) house (0.005) law (0.005) job (0.004) situation (0.004) thing (0.004)
6	Schools and infection	0.10	school (0.02) time (0.014) news (0.014) lot (0.011) mask (0.011) day (0.011) year (0.01) video (0.009) student (0.009) China (0.007) Corona (0.007) Seoul (0.007) money (0.006) comment (0.006) Korea (0.006) government (0.006) lecture (0.005) place (0.005) country (0.005) bus (0.005) today (0.005) information (0.004) company (0.004) home (0.004) house (0.004) child (0.004) area (0.004) YouTube (0.004) Wuhan (0.003) dong (0.003)
7	Political leader	0.10	Korea (0.021) China (0.02) country (0.013) Corona (0.013) government (0.011) mask (0.008) Fighting (0.008) time (0.007) comment (0.007) Trump (0.007) dog (0.007) haha (0.007) Daegu (0.007) Moon (0.007) student (0.006) Jae (0.006) child (0.005) news (0.005) world (0.005) Jinping (0.005) guy (0.005) lot (0.005) video (0.005) Wuhan (0.005) lol (0.005) Xi (0.004) thing (0.004) everyone (0.004) citizen (0.004) staff (0.004)
8	Religion, politics, and infection	0.09	God (0.018) Kim (0.017) church (0.016) Park (0.011) Lee (0.009) pastor (0.009) time (0.009) Corona (0.008) word (0.007) worship (0.006) hoon (0.006) Jae (0.006) life (0.006) China (0.005) Lord (0.005) hyun (0.005) country (0.005) won (0.005) Kwang (0.005) Jeon (0.005) Jesus (0.004) Moon (0.004) world (0.004) min (0.004) Jang (0.004) lot (0.004) Choi (0.004) Hyun (0.004) Min (0.004) Jung (0.004)

## Discussion

In previous sections, we described our analysis of YouTube data from the early days of the COVID-19 outbreak in South Korea to illustrate the social construction of cognition and emotion. Our analysis revealed that social cognitive appraisals of COVID-19-related events were linked to fluctuations in fear and anger emotions, indicating that fear and anger can be understood as separate and distinct emotions, offering insights into their underlying triggers. Our study results support the following discussion points.

Two key factors emerged during the early stages of the COVID-19 pandemic. The first was the scarcity of accurate medical and health information accessible to the public regarding the outbreak and the infectious disease. Additionally, the rapid spread of infections made it challenging for healthcare institutions to react promptly. The second factor was the absence of a vaccine and cure, which prompted the implementation of non-pharmaceutical measures like social distancing to manage the pandemic [41]. These features highlighted the connection between the success of the pandemic control policy and the social attitudes of citizens toward the government. This underscores the importance of public health in the early stages and citizen participation in the policy.

During the COVID-19 pandemic, there was a noticeable pattern in the order of emotional responses to the situation. Fear saw a rise and fall, with anger then taking its place. In January 2020, fear was the predominant emotion, followed by anger in March. By August, fear began to climb steadily, reaching its peak in September before descending, while anger escalated more rapidly than fear. However, in the following months, anger decreased as fear mounted. By December, anger diminished as fear intensified. The period between June and August stood out as distinct, with anger skyrocketing while fear experienced a slower incline.

As shown in Fig. 3, the changes in fear and anger aligned with significant social events related to COVID-19 [24, 26]. First, fear emerged in January 2020, peaked in February, and began to decline, followed by anger in March. During this time, fear remained relatively low since the outbreak began in Wuhan, China, in December 2019 until 20 January 2020, when the first domestic cases were confirmed. Anger also stayed at a relatively low level. Media reports suggested edible wildlife like bats, pangolins, and minks sold at Wuhan’s Huanan Market on 23 January were the source of the outbreak but were not seen as a direct threat to South Korean society, as it was only linked to an unsanitary food culture [42]. Fear reached its peak on 18 February 2020, when around 5,000 individuals were infected in Daegu and Gyeongsangbuk-do, one month after the initial case was identified. During this period, there was a scarcity of precise information regarding COVID-19. Subsequent investigations conducted by the Korea Centres for Disease Control and Prevention and law enforcement uncovered the extent of the involvement of the Shincheonji Church and the rising social unrest among citizens [43]. This corresponded to the first wave of the COVID-19 pandemic.

In August 2020, Love First Church and Gwanghwamun political rallies were reported as threats to the spread of the infection. Analysis of comments during that time showed strong anger toward far-right Christians and fears of outbreaks around August 15. The number of citizens diagnosed with COVID-19 increased by nearly 1,000 in August. Social anger decreased in September as the threat of the Gwanghwamun Rally diminished. Fear and anger rose from July to September, corresponding to the second wave of the COVID-19 pandemic.

In November and December, outbreaks rapidly spread across the country, particularly in large cities, including nursing homes, religious institutions, businesses, and educational facilities. Unlike February, social fears were relatively low, possibly due to the availability of professional medical information about the pandemic and the systematic promotion of pandemic prevention policies by infectious disease control authorities. However, fear levels started to rise slightly toward the end of December, marking the beginning of the third wave of the pandemic. Social anger exhibited similar fluctuations, corresponding to the third wave of the COVID-19 pandemic.

The cause of social fear and citizens’ emotional moods in May were unique. Fear was intense initially and then varied gradually. The initial issue seemed to be the lack of accurate information about COVID-19, which affected the intensity of the social threat [44]. In May 2020, there was a sudden increase in social anger as fear decreased, possibly linked to the well-controlled Itaewon outbreak. The outbreak reduced social fear but raised ethical issues due to some young people violating quarantine protocols, leading to social anger. Reports also indicated that the infection source in the Itaewon case was a homosexual, triggering social disgust and prejudice.

Using LDA topic modeling, eight representative topics were derived and labeled. These topics reflected citizens’ interests and social attitudes toward coronavirus-related events in the first year of COVID-19.

The initial topic discussed was “causes and risks.” The pertinent comments about this topic focused on blame for the pandemic outbreak, specifically the infectious disease that originated in Wuhan, China [45]. Comments were made about bats, animals, mortality, and problems, specifically addressing social attitudes criticizing the morality of zoonotic infections. These assessments greatly worried citizens and seemed to be a significant cause of social fear and anger.

The second topic, “perception of China,” included feedback from individuals in China, Korea, Japan, and the United States. It reflected citizens’ interest in global quarantine policies and vaccine development, indicating that people viewed COVID-19 as a political and economic concern, not solely a health issue.

The third topic, “media and information,” focused on COVID-19 updates. Unlike previous outbreaks, the daily dissemination of COVID-19-related data by government health authorities, such as case numbers, death tolls, prevention guidelines, and interviews with medical professionals, was perceived to impact people’s social attitudes. This information could either decrease or increase feelings of fear and anger, subsequently influencing behavior like social distancing.

The fourth topic, “infection prevention rules,” was a significant concern for people both at home and around the world. South Korea successfully enforced social distancing early on with active citizen participation, making it a crucial topic for the public.

The fifth topic was “economic activity.” The ongoing social distancing measures implemented in phases in South Korea led to the direct or indirect isolation of citizens. As a result, production and consumption activities were limited, highlighting worries about the financial well-being of individual citizens rather than the overall state of the national economy.

The sixth topic was “schools and infection,” which underscored concerns about the transmission of the virus in educational settings, particularly among young people who struggled to adhere to quarantine protocols during the early stages of the pandemic when herd immunity was low. This trend was emblematic of South Korean society, where education holds great significance.

The seventh topic was “political leader,” and the eighth was “religion, politics, and infection.” During the COVID-19 pandemic, political leaders’ stances on preventive policies caused concern. Citizens often mentioned political leaders, particularly in relation to the second wave of the pandemic starting in August. This wave was attributed to overzealous collective action by religious organizations with conservative political leanings [46]. Religious organizations were blamed for frequent outbreaks that violated quarantine rules, contributing to increased social anger.

This study has several implications. First, the emergence of COVID-19 as a global threat has prompted rapid research in various healthcare fields worldwide. However, existing robust approaches in the clinical field are limited to identifying and solving problems quickly and efficiently in complex pandemic situations [47]. Therefore, we believe that conducting a study to quantitatively analyze trends in negative social emotions during a pandemic related to recent events would be beneficial in supplementing clinical research.

Second, the analytical method utilized in this study quantitatively analyzed networks and topic themes formed by interactions and relationships among keywords. This enabled us to categorize the existing COVID-19 research broadly, in order to investigate the knowledge structure and monitor the evolution of research topics over time. This information can be utilized to further explore related research topics in the future.

Large-scale infectious diseases, such as COVID-19, are closely connected to psychological issues within social communities. Psychological distress in COVID-19 patients has been linked to feelings of guilt, helplessness, fear of the unknown progression of the disease, and symptoms of depression [48, 49]. During the COVID-19 pandemic, mental health had been affected in patients and healthcare workers on the frontline. Many of them experienced mood and sleep disturbances, leading to mental health risks [50]. Psychosocial interventions must be accompanied by aggressive COVID-19-related physical treatments.

The study analyzed the relationship between social-emotional conditions and the clinical crisis of COVID-19. This interdisciplinary study highlights that large-scale infectious diseases are influenced by genetic and biological factors, as well as social, political, economic, and ecological factors [51].

Based on the pandemic outbreaks in the 2000s, it is probable that another large-scale infectious disease outbreak will occur soon. The initial stages of the outbreak are likely to resemble those of the COVID-19 outbreak in 2020. Social disruption and citizens’ emotional responses are expected to be crucial. Therefore, this study, which analyzes the social cognitive factors influencing the social emotions of citizens during the early stages of such outbreaks, can aid in shaping pandemic prevention and public health policies for similar scenarios.

Our study aimed to systematically present the social factors that healthcare policies should consider more carefully to help citizens whose lives and health were threatened during the COVID-19 pandemic in South Korea. It is also expected to assist healthcare professionals in responding effectively to future outbreaks. Recent studies have suggested that healthcare professionals should focus on the social and psychological factors of citizens to improve responses to large-scale outbreaks. Widespread infection and extended isolation times lead to various social problems, necessitating healthcare policies that gain the consensus and agreement of citizens. Our analysis of social factors in pandemic situations focused on citizens’ cognitions and emotions. Building on the cognitive-emotional theory, we explored the correlation between distinct cognitive evaluative factors and emotions. While other studies aim to explain cognitions and emotions in pandemics, they often fail to distinguish between individual and social characteristics of cognition and emotion, resulting in a vague presentation of negative emotions. By contrast, our study provides a more systematic understanding of the social aspects of negative emotions within the pandemic context.

We must take wise action against recurring pandemic diseases like Middle East respiratory syndrome, COVID-19, and others. Particularly, since people’s social emotions during a pandemic impact their attitude and behavior in overcoming it, it is crucial to address social and psychological quarantine, which may be neglected when treating physical illnesses.

In the first year of the COVID-19 outbreak, anxiety initially increased and then stabilized, while anger gradually increased. Immediate and proactive responses are essential to successfully decrease anxiety and anger, thus demanding effective national and community efforts.

As psychological problems can impact other biological factors based on symptom severity, it is believed that active COVID-19 personalized psychosocial interventions are necessary. Instead of waiting for individuals to seek support when they reach a breaking point, it is vital to provide proactive services by predicting when they may be needed and delivering them through real-time monitoring and analysis. Various IT platforms, like interactive agent robots and digital therapeutics for counseling and psychotherapy [52–54], should be utilized to constantly monitor changes and facilitate immediate communication and response.

YouTube comments often directly express viewers’ emotions and feelings about a video, with both positive and negative reactions. Under these conditions, it is necessary to extract adjectives, adverbs, and other spoken parts of speech in addition to nouns when identifying emotional lexemes in comments.

On the other hand, the comments are often short or single-word in nature, with a significant proportion of simple reactions or sentiments expressed in one or two sentences. Therefore, the actual content may seem shorter than the comment length indicates. In addition, comments often use colloquialisms, abbreviations, and emoticons, which often omit punctuation and spaces. Due to these features, our study focused on nouns to extract emotional words when analyzing YouTube comments. However, this is different from other studies of emotion-cognition theories that mainly analyze adjectival emotional lexemes, which could act as a limitation of our study. Furthermore, our study’s approach of combining noun-focused analysis, LDA, and semantic network analysis with emotion analysis has not been extensively explored in the existing literature, as far as we have been able to ascertain.

However, despite these limitations, we believe that our approach can contribute to the field by providing a unique perspective on YouTube comment analysis, and in future work, we hope to address these shortcomings by exploring alternative methods for corpus construction and sentiment analysis that can better capture the nuances of the original language.

Cultural differences and their potential impact on emotion analysis are classic and controversial issues of emotion-cognition theories. Depending on the researchers, cultural differences in emotions can be either positive or negative, which several researchers struggle with, and depending on the conditions of the study, translation and researcher interpretation are important aspects [55]. Unfortunately, this was also a limitation in our study. We acknowledge that the lack of a comprehensive Korean emotion lexicon is a limitation of our study, leading us to utilize the NRC Emotion Lexicon (EmoLex) for our analysis.

The NRC Emotion Lexicon is based on the English language, which may not fully capture the cultural nuances of emotions expressed in Korean. However, it is worth noting that the NRC lexicon has been widely used in sentiment analysis studies across various non-English speaking contexts. The creators of the NRC Emotion Lexicon have stated that, despite some cultural differences and errors, the lexicon maintains sufficient validity when translated into 108 languages.

Nonetheless, we recognize that the reliance on a translated lexicon may have introduced some limitations in our analysis. We believe that the development of more sophisticated Korean sentiment analysis tools in the future will greatly benefit research in this area.

Our study limitations include the following. First, the broad nature of the networks we derived suggests the need for further fine-grained cross-sectional and longitudinal analyses. Second, there should be a specific methodological agreement on the process of labeling topics. In the future, we aim to address these gaps and contribute to public health research. Third, our focus was on social fear and anger during the pandemic. However, other negative emotions such as disgust and anxiety were also present in some of our data analyses. Future studies may allow us to complement these social cognitive-emotional dimensions. Finally, the keyword network from this study was extensive, including overall term relationships. Therefore, a detailed analysis focusing on key keywords will be necessary in future research.

## Conclusions

This study confirmed that the COVID-19 pandemic, along with negative emotions, was associated with other cognitive conditions that impacted the development of social attitudes. Our research is significant as it aimed to pinpoint key themes in comments about COVID-19 on social media, particularly focusing on fear and anxiety stemming from major social or political events. Our findings can be valuable for future efforts in education, research, and practical interventions to prevent and address new pandemics post-COVID-19.

## Data Availability

All data analyzed during this study are included in the published comments. The datasets are available from the corresponding author upon reasonable request.

## Abbreviations

LDA: Latent Dirichlet Allocation
NRC: National Research Council
POS: Part-of-speech
TDM: Term-document matrix
